# Tigecycline and Gentamicin-Combined Treatment Enhances Renal Damage: Oxidative Stress, Inflammatory Reaction, and Apoptosis Interplay

**DOI:** 10.3390/ph15060736

**Published:** 2022-06-10

**Authors:** Dina Elgazzar, Mohamed Aboubakr, Heba Bayoumi, Amany N. Ibrahim, Safwa M. Sorour, Mohamed El-Hewaity, Abulmaaty M. Elsayed, Shaimaa A. Shehata, Khaled A. Bayoumi, Mohammed Alsieni, Maged Behery, Doaa Abdelrahaman, Samah F. Ibrahim, Ahmed Abdeen

**Affiliations:** 1 Department of Veterinary Pharmacology, Faculty of Veterinary Medicine, Benha University, Toukh 13736, Egypt; mohamed.aboubakr@fvtm.bu.edu.eg; 2 Histology and Cell Biology Department, Faculty of Medicine, Benha University, Benha 13518, Egypt; heba.bayoumi@fmed.bu.edu.eg; 3 Department of Pharmacology, Faculty of Medicine, Benha University, Benha 13518, Egypt; amani.ibrahim@fmed.bu.edu.eg (A.N.I.); safwasorour81@gmail.com (S.M.S.); 4 Department of Veterinary Pharmacology, Faculty of Veterinary Medicine, Menoufia University, Shebin Elkoum 32514, Egypt; melhewaty82@gmail.com; 5 Anatomy and Histology Department, Faculty of Medicine, Mutah University, Mutah 61710, Jordan; abulsala@mutah.edu.jo; 6 Department of Anatomy and Embryology, Faculty of Medicine, Benha University, Benha 13518, Egypt; maged.behery@fmed.bu.edu.eg; 7 Department of Forensic Medicine and Clinical Toxicology, Faculty of Medicine, Suez Canal University, Ismailia 41522, Egypt; shaimaa_shehata@med.suez.edu.eg; 8 Department of Forensic Medicine and Clinical Toxicology, Faculty of Medicine, Cairo University, Cairo 11956, Egypt; kabadr@kau.edu.sa; 9 Department of Pathology, Faculty of Medicine, King Abdulaziz University, Jeddah 21442, Saudi Arabia; 10 Department of Pharmacology, Faculty of Medicine, King Abdulaziz University, Jeddah 21442, Saudi Arabia; malsieni@kau.edu.sa; 11 Department of Clinical Sciences, College of Medicine, Princess Nourah bint Abdulrahman University, P.O. Box 84428, Riyadh 11671, Saudi Arabia; dsabdelrahman@pnu.edu.sa (D.A.); sfibrahim@pnu.edu.sa (S.F.I.); 12 Department of Forensic Medicine and Toxicology, Faculty of Veterinary Medicine, Benha University, Toukh 13736, Egypt; 13 Center of Excellence for Screening of Environmental Contaminants (CESEC), Benha University, Toukh 13736, Egypt

**Keywords:** tetracyclines, aminoglycosides, kidney injury, oxidative damage, annexin-V, inflammatory cytokines, PCNA

## Abstract

Although the combination of antibiotics is generally well-tolerated, they may have nephrotoxic effects. This study investigated whether tigecycline (TG) and gentamicin (GM) co-administration could accelerate renal damage. Male Wistar rats were randomly divided into six experimental groups: the control, TG7 (tigecycline, 7 mg/kg), TG14 (tigecycline, 14 mg/kg), GM (gentamicin, 80 mg/kg), TG7+GM, and TG14+GM groups. The combination of TG and GM evoked renal damage seen by the disruption of kidney function tests. The perturbation of renal tissue was mainly confounded to the TG and GM-induced oxidative damage, which was exhibited by marked increases in renal MDA (malondialdehyde) along with a drastic reduction in GSH (reduced-glutathione) content and CAT (catalase) activity compared to their individual treatments. More obvious apoptotic events and inflammation were also revealed by elevating the annexin-V and interleukin-6 (IL-6) levels, aside from the upregulation of renal PCNA (proliferating cell nuclear antigen) expression in the TG and GM concurrent treatment. The principal component analysis indicated that creatinine, urea, annexin-V, IL-6, and MDA all played a role in discriminating the TG and GM combined toxicity. Oxidative stress, inflammatory response, and apoptosis were the key mechanisms involved in this potentiated toxicity.

## 1. Introduction

**Figure 1:**
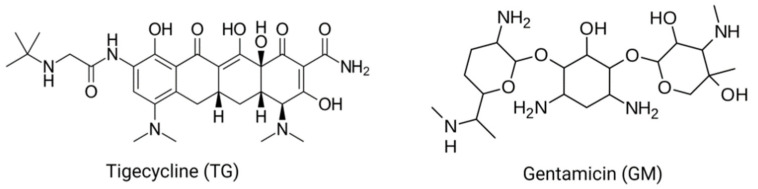
The chemical structure of tigecycline and gentamicin.

Tigecycline (TG) is a newly emerged broad-spectrum antibiotic that belongs to the glycylcycline class, which is a synthetic analog to tetracyclines. It was approved by the Food and Drug Administration (FDA) in 2005. TG is structurally and functionally mostly related to tetracyclines but with more potent activity against tetracycline-resistant pathogens [1,2,3]. The addition of an *N*,*N*,-dimethylglycylamido group at the 9 position of the minocycline molecule increases the affinity of TG for the ribosomal target up to five times when compared with tetracycline (Figure 1). This allows for an expanded spectrum of activity and decreased susceptibility to the development of resistance. Alterations to the tetracycline structure allow for TG to maintain activity against tetracycline-resistant organisms including resistant Gram-positive organisms [4,5,6]. Based on this therapeutic importance, TG has gained more attention among the other antibiotics indicated for a variety of infectious diseases including lung, abdominal, and skin infections [7,8]. In contrast to the currently marketed tetracyclines, which are accessible in oral dose forms, TG is exclusively available in an injectable formulation for clinical use. It has a much larger distribution volume than the other tetracyclines. TG is reported to have a short half-life, and thus is injected daily for about 14 days to exert its pharmacological action [9].

Aside from the antibacterial potential of TG, it has recently exhibited antitumor activity against many types of tumors. TG has shown the ability to inhibit cell proliferation and metastasis in various types of cancer such as human acute myeloid leukemia [10], melanoma [1], gastric cancer [11], oral squamous cell carcinoma [12], and neuroblastoma [13]. Mitochondrial oxidative damage, inhibition of the cell cycle, and induction of apoptosis are the main mechanisms associated with the cytotoxic effect of TG [14,15,16].

Despite TG’s therapeutic considerations, Hu et al. [8] and Lin et al. [17] reported the development of hypofibrinogenemia and acute pancreatitis in patients receiving TG, respectively. Li et al. [18] documented the patients’ vulnerability with liver and kidney affections to the TG toxicity. Indeed, the available literature regarding the toxic mechanistic actions associated with the TG regimen when given alone or in combination is very scanty. This highlights the particular interest of exploring the mechanisms behind TG-induced toxicity.

Gentamicin (GM; Figure 1) is an aminoglycoside antibiotic that has strong efficacy against Gram-negative microorganisms that cause severe infections in humans and animals [19]. However, it has a significant disadvantage because it causes ototoxic and nephrotoxic consequences [20]. GM remains an effective medicine to battle strains of microorganisms that have evolved resistance to multiple antibiotics, despite the associated side effects. However, in developing countries, the use of GM is common because of its availability, effectiveness, and cost [21]. The GM-induced nephrotoxicity is triggered via pathological mechanisms connected with apoptosis, oxidative stress, inflammation, and necrosis [19,22,23,24,25].

Furthermore, TG and GM have a pharmacodynamically synergistic therapeutic impact in maintaining and improving efficacy against a broad range of pathogens since both bind to the same sites on the 30S ribosomal subunit in the bacterial cell [26]. In an animal model of infected endocarditis, both antibiotics had a strong antibacterial impact [27]. Moreover, a combined therapy of TG and GM has shown a potent bactericidal activity against *Klebsiella pneumoniae* infections in trauma intensive care unit patients without other comorbidities [28]. So far, to the best of our knowledge, the nephrotoxic effect of TG when given alone or in combination with GM has not been investigated. Due to these, the current work was postulated to observe the renal function, oxidative injury, apoptosis, and inflammatory response induced by TG and/or GM treatment.

## 2. Results

### 2.1. Effect of TG and GM Combination on the Kidney Function

**Figure 2:**
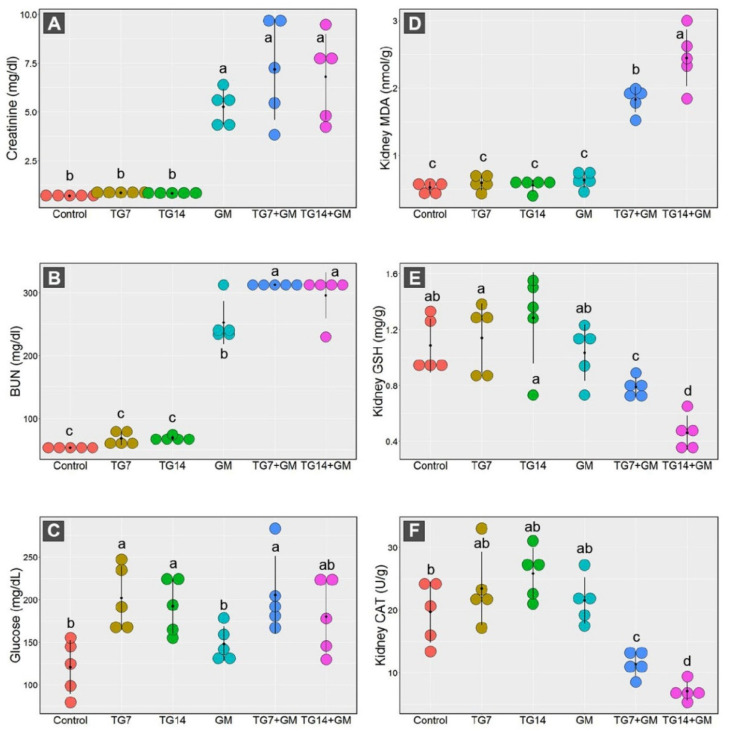
Dot plot panel with the means (black dot) and 95% confidence interval (the stretching out black lines from the means) of the creatinine (**A**), BUN (blood urea nitrogen) (**B**), glucose (**C**), MDA (malondialdehyde) (**D**), GSH (reduced-glutathione) (**E**), CAT (catalase enzyme) (**F**) in the control, TG7 (tigecycline, 7 mg/kg), TG14 (tigecycline, 14 mg/kg), GM (gentamicin), TG7+GM, and TG14+GM groups. The different lower case letters are considered statistically significant at *p* ≤ 0.05.

The dot plot presented in Figure 2 showed dramatic increases in the serum levels of creatinine and BUN in animals treated with GM alone and those receiving GM with TG low and high doses (TG7+GM and TG14+GM, respectively) in comparison to the other groups. However, the glucose levels recorded significant increases in animals treated with TG alone and those treated with a combination of TG and GM compared to the controls.

### 2.2. Effect of TG and GM Combination on the Oxidative State of Renal Tissue

Our data framed the existence of marked disruption in the oxidative state of the renal cells in response to the combined treatment of both TG and GM. As depicted in Figure 2, the renal MDA levels were greatly increased after the administration of TG and GM in combination compared to the control, TG7, TG14, and GM groups. By the same pattern, the co-administration of TG and GM could substantially reduce the GSH concentration levels along with a marked decrease in the CAT activity in renal tissue.

Notably, the alterations in the renal antioxidant components induced by combined treatment of TG and GM were dose-dependent. As seen, the combination of GM with the higher dose of TG (14 mg/Kg) evoked more damage than those that received GM with the lower dose of TG (7 mg/Kg).

### 2.3. Effect of TG and GM Combination on Apoptosis and Inflammatory Response

**Figure 3:**
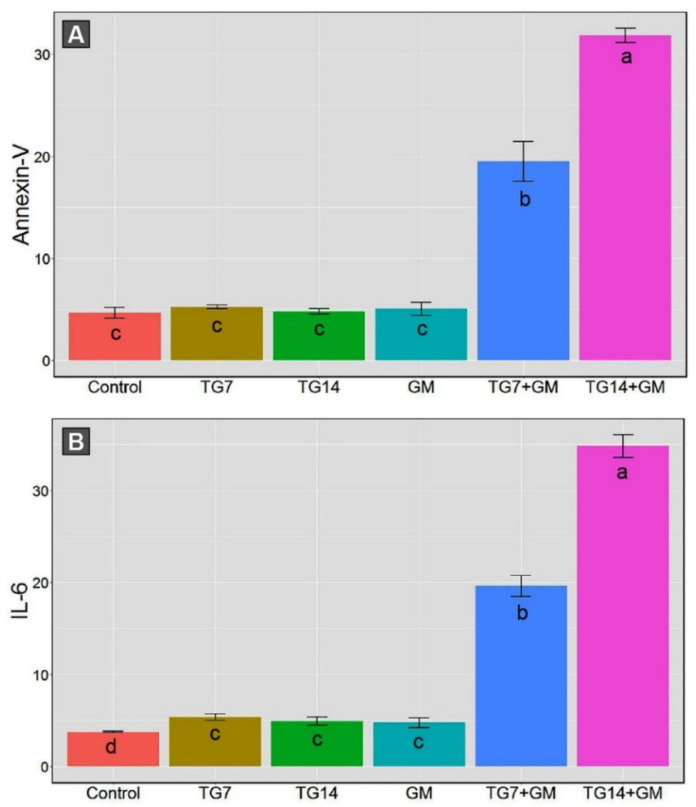
The bar plot panel with the means and 95% confidence interval of the kidney apoptotic cell obtained from annexin-V (**A**) and the tissue levels of the inflammatory cytokine (IL-6) (**B**) in the control, TG7 (tigecycline, 7 mg/kg), TG14 (tigecycline, 14 mg/kg), GM (gentamicin), TG7+GM, and TG14+GM groups. The different lower case letters are considered statistically significant at *p* ≤ 0.05.

The data obtained from the annexin-V analysis demonstrated that the proportions of intact cells were drastically decreased, along with significant increases in the apoptotic cells (early and late) and necrotic cells in animals that received both TG and GM in comparison to their sole treatments (Figure 3). Within the framework of the data above-mentioned, the co-administration of GM with 14 mg/kg TG (TG14+GM group) triggered more apoptotic sequels than the other corresponding combinations with a 7 mg/kg TG (TG7+GM group).

Moreover, the combined therapy of TG and GM initiated the inflammatory reactions indicated by an increase in the tissue levels of the inflammatory cytokine (IL-6), as illustrated in Figure 3. The renal IL-6 levels were substantially enhanced in the TG7+GM and TG14+GM groups compared to the control, TG7, TG14, and GM groups. Despite the promoted inflammatory reaction in both doses of combined therapies, the intensity of the inflammation was lesser in the TG7+GM group than the TG14+GM group. These data suggest that the effect of the combined treatment of TG and GM occurred in a dose-dependent pattern.

### 2.4. Histopathological Changes in Kidney Tissue

**Figure 4:**
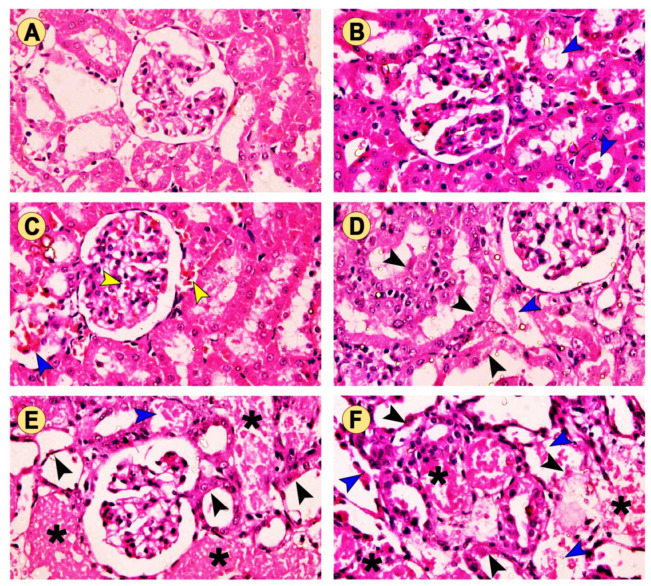
The effect of TG and GM combined treatment on the renal histoarchitecture. (**A**) The control group presented normal renal corpuscle and proximal and distal convoluted tubules. (**B**) The TG7 group showed mild intertubular eosinophilic debris (blue arrow) in the lumina of some renal tubules. (**C**) The TG14 group showed congestion of the glomerular tufts and intertubular blood capillaries (yellow arrow) as well as mild intratubular eosinophilic debris (blue arrow). (**D**) The GM group exhibited moderate intratubular eosinophilic debris (blue arrow) and degenerated tubular epithelium (black arrow). (**E**,**F**) The TG7+GM and TG14+GM groups, respectively, showed severely degenerated tubules with an accumulation (asterisk) of dead tissues within their lumina in a dose-dependent manner (H&E stain, 400× magnification).

For further confirmation of the data described above, a histopathological examination was performed to evaluate the changes in the kidney architecture after treatment with TG and/or GM. As depicted in Figure 4A, the control animals exhibited a normal renal architecture displayed by normal renal corpuscle, renal convoluted tubules (proximal and distal), and collecting ducts. In contrast, rats treated with TG alone had degenerative changes in the renal glomeruli, but non-significant changes in the tubules associated with tubular vacuolization (Figure 4B,C). The TG7 group showed mild inter-tubular eosinophilic debris in the lumina of some renal tubules and some apoptotic renal cells. Furthermore, the TG14 group revealed the congestion of the glomerular tufts, inter-tubular blood capillaries, and mild intratubular eosinophilic debris. However, the GM-treated group exhibited a moderate amount of intratubular eosinophilic debris and degenerated tubular epithelium compared to those seen in the TG7 and TG14 groups (Figure 4D). Expectedly, the renal tubules of both the TG7+GM and TG14+GM groups demonstrated significant severe degeneration, the accumulation of dead tissues in their lumina, and congestion of the interstitial blood vessels (Figure 4E,F). Interestingly, the scoring summary of the renal histopathological changes suggested that the effect of the combination (TG and GM) caused more damage in the renal tissue in a dose-like manner (Table 1). More images for renal histopathology after TG and/or GM treatment are also presented in Figure S1 (Supplementary Materials).

### 2.5. Effect of TG and GM Combination on PCNA Protein Expression Level

**Figure 5:**
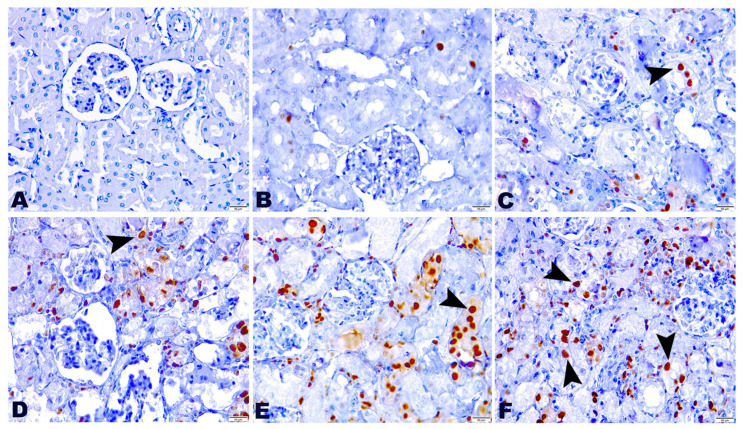
The effect of TG and GM combined treatment on the PCNA expression in renal tissue. (**A**) The control group exhibited negative PCNA expression. (**B**,**C**) The TG7 and TG14-treated groups pronounced few nuclear PCNA reactions. (**D**) The GM group bared moderate reactivity to PCNA. (**E**,**F**) The TG7+GM and TG14+GM groups highlighted intense positive nuclear PCNA expression in a dose-dependent manner. The positive staining of PCNA is pointed out by the arrows and indicated by a brown stained renal nuclei (PCNA, 400× magnification).

The presented work indicated the existence of DNA damage and repair mechanisms induced by TG and/or GM treatment characterized by the increased expression of PCNA. The detection and distribution of PCNA protein expression in the kidney tissues after treatment with a combination of TG and GM at two dose levels are presented in Figure 5. TG and GM, when given alone or in combinations, could obviously enhance the PCNA expression in renal cells, as seen by the increased number of positive nuclei compared to the controls. However, the upregulation of PCNA expression was more evident in the animals that co-administrated TG and GM compared to the other treated groups. These findings support the concept that combined therapy of both agents used in the current work was able to substantially upregulate the PCNA in a dose-dependent manner, confirming the histopathological data.

### 2.6. Principal Component Analysis (PCA)

**Figure 6:**
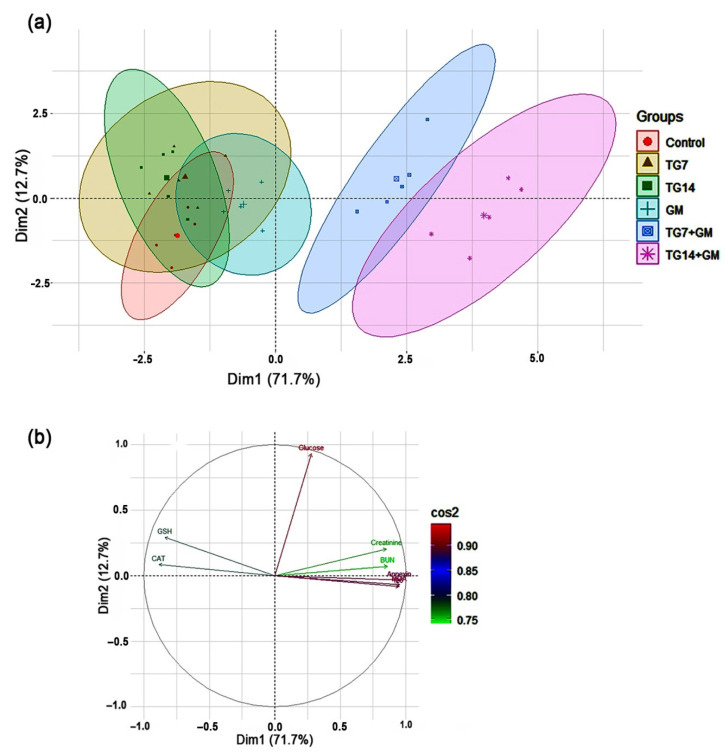
The principal component analysis (PCA) biplot. (**a**) The PCA for thirty treated rats, the control (red circles, *n* = 5), TG7 (tigecycline, 7 mg/kg; *n* = 5; yellow triangles), TG14 (tigecycline, 14 mg/kg; *n* = 5; green squares), GM (gentamicin; *n* = 5; blue pluses), TG7+GM (*n* = 5; blue squares), and TG14+GM (*n* = 5; pink asterisks). Percentage values indicated on the axes represent the contribution rate of Dim1 and 2 to the total amount of variation. (**b**) The contribution degree of the different variables. The colored scale indicates that the strength of the contribution ranged from the highest (red color) to lowest contribution (green color).

All of the investigated parameters (creatinine, BUN, glucose, MDA, GSH, CAT, annexin-V, IL-6) were loaded into two major dimensional components (Dim1 and Dim2), explaining 84.4% of the total variance. Most of the examined parameters were discriminated by Dim1, and thus described by the larger proportion of variance (71.7%), while the lower proportion of variance (12.7%) was captured by Dim2 (Figure 6). The PCA score plot revealed two distinct groups, where the groups receiving combined therapy (TG7+GM and TG14+GM) were clustered together and separated from those receiving individual treatments (control, TG7, TG14, and GM) (Figure 6a). In the PCA loading plot, creatinine, BUN, MDA, annexin-V, and IL-6 were positively associated with the TG7+GM and TG14+GM groups. On the other hand, GSH and CAT were positively linked with the control, TG7, TG14, and GM groups (Figure 6b).

## 3. Discussion

TG is a newly introduced broad-spectrum antibiotic among the family glycylcycline descended from family tetracyclines. It approved its efficacy against tetracycline-resistant bacteria aside from its anticancer activity [2,4,5]. On the other hand, several studies have shown a variety of drawbacks associated with the TG regimen including renal insufficiency [18], pancreatitis [17], and hypofibrinogenemia [8]. GM is an aminoglycoside antibiotic that has a potent bactericidal effect on Gram-negative bacteria with a well-known nephrotoxic demerit [21]. Co-administration of TG with some other antibiotics such as GM [27], rifampicin [29], and vancomycin [30] have been appreciated by researchers in order to potentiate the efficacy of TG against resistant-pathogen related diseases. It is well-known that kidneys are easily susceptible to damage from drug burden because of larger perfusion and increased concentration of excreted drugs along the renal tubules. Due to these, the combined therapy of both TG and GM is supposed to burden the kidney function ability. As seen in the current study, combined treatment of TG and GM could evoke a renal damage indicated by elevated serum creatinine, BUN, and glucose levels. Although the serum creatinine is a more accurate marker than BUN, it was not altered in the TG and GM combination in comparison to GM alone. The fact that TG alone and TG+GM both generated significant increases in the blood glucose levels when compared to the controls or the GM-treated group was most likely due to TG-induced pancreatitis. Furthermore, severe diarrhea is a common adverse effect of TG, leading to dehydration [17]. This could be another reason for the elevated BUN levels without any alterations in the creatinine levels.

Despite the shortage of the available data regarding the impact of TG on renal tissue, Dong et al. [2] suggested the implication of TG in mitochondrial dysfunction, oxidative damage, and apoptosis in different cancer cell lines. Moreover, there is a strong body of evidence supporting the involvement of oxidative stress and apoptotic cascade in GM-induced renal injury [19,31,32,33].

In a state of imbalance between free radicals and cellular detoxifying mechanisms (enzymatic and non-enzymatic antioxidant components), oxidative distress is known to begin. The major manifestations of oxidative distress in the biological system include lipid peroxidation, mitochondrial dysfunction, reduced ATP production, protein misfolding, and DNA oxidation [34]. It is well-known that during oxidative damage, the overproduction of reactive oxygen radicals (such as O_2_^•−^, H_2_O_2_, OH^•^, and NO) are generated; thereby, GSH (non-enzymatic antioxidant) and CAT (enzymatic antioxidant) are depleted. The current analysis consistently indicated the presence of a disrupted oxidative state after combined treatment with TG and GM, while no changes were observed after their solitary therapy. These findings are indicated by a substantial reduction in the GSH levels and CAT activity in renal tissue in the combined therapies only. CAT functions in catalyzing the generated H_2_O_2_ into H_2_O and O_2_. It is well-documented that when CAT is defeated, a highly reactive OH^•^ is produced via an iron-catalyzed Haber–Weiss reaction (H_2_O_2_ + Fe^2+^ → OH^•^ + OH^−^ + Fe^3+^) [35,36]. OH^•^ can remotely attack the cellular molecules, mainly the lipid content of the cell membranes, causing lipid peroxidation and the further production of another harmful substance (namely, MDA), which makes the matter worse [37,38]. In response to the TG and GM combined-insult, our data consistently showed a drastic increase in the MDA levels in the kidney tissue. However, there were no recorded alterations in the MDA concentrations after treatment with the TG or GM alone. Despite the fact that oxidative damage has been implicated in the toxic mechanisms of TG and GM [19,31,32,33], our modified model demonstrated no changes in the biochemical studies of the cellular oxidative state after treatment with TG or GM alone. These findings might indicate the initiation of an endogenous antioxidant compensatory response and downstream adaptations to protect those animals from oxidative damage [39]. Unlike the oxidative stress, IL-6, and annexin-V data, it was noticed that the serum creatinine and BUN levels were elevated in the GM-exposed animals, which might also be attributed to the latter reason. Moreover, Lin et al. documented that TG was able to induce pancreatitis, which contributed to the TG-observed hyperglycemia in our study [17]. Regardless of the oxidative stress-mediated renal injury, the reported TG-induced hyperglycemia would be another possible cause for the accelerated damage that occurred during the combined therapy regimen.

Along with the above-mentioned findings, the histopathological examination exhibited increased intratubular eosinophilic debris that might be attributed to the sloughing of the renal apical membrane into the tubular lumen, supporting the concept that lipid peroxidation plays a crucial role in modulating the TG and GM combined therapy-induced renal injury. Tubular degeneration was also observed in our study that would have occurred as a consequence of generalized damage caused by the accumulation of the harmful radical. A growing body of evidence reported that oxidative species are implicated in the disruption of the nephron’s excretory activity, resulting in a homeostasis imbalance and the accumulation of metabolic products [40,41,42]. It is well-known that mitochondria are highly abundant in renal cells, offering plenty of ATP required for active transport and the elimination of metabolic byproducts. Meanwhile, these damaging radicals caused the inhibition of the mitochondrial oxidative phosphorylation necessary for ATP production [34,43].

Therefore, we assumed that the existing renal damage might be attributed to the oxidative damage induced by the combined administration of TG and GM.

OH^•^ and MDA have been reported to directly bind to guanine bases in the genomic DNA, forming guanine adducts and DNA damage [44]. Thus, the created MDA combined with the produced oxidative radicals amplified the deleterious effects of TG and GM co-treatment. These damaging oxidants contributed to the increased mitochondrial membrane permeability, followed by the release of cytochrome c, causing the protein oxidation and aggregation of unfolded proteins, and the activation of NF-kB and, ultimately, the induction of an apoptotic cascade [34,41,45]. In the current study, the increased levels of annexin-V after co-treatment with both TG and GM suggest that these events activate the apoptotic cascade. Apoptosis has also been linked to changes in the GSH redox status caused by the oxidation of glutamine, a precursor for GSH production [46]. PCNA is a nuclear protein that plays a key function in DNA replication and repair [47]. This study clarified that a marked increase in immunoreactivity against PCNA, which could be a reaction to the TG and GM-inflicted DNA damage. These data are in the same line as our previous studies that reported upregulated PCNA in oxidative stressed and DNA damaged-cells after different intoxication with acetaminophen [40] and aflatoxin B_1_ [42]. The renal histopathology revealed a generalized tubular degeneration confirming the above-mentioned findings and supporting the existence of a progressive renal dysfunction in groups treated with TG and GM simultaneously when compared to their sole treatments.

In the current investigation, the inflammatory cytokine, IL-6, was notably elevated in TG and GM co-administrated animals in renal tissue. Oxidative stress is linked to intracellular signaling including the activation of NF-kB. Thereby, the proinflammatory genes are upregulated; hereby, the inflammatory cytokines are extensively excreted, leading to the occurrence of the inflammatory response, which contributed to renal injury [34]. Our team has previously recorded enhanced inflammatory reactions in response to chlorpyrifos and microcystin-induced oxidative damage in cerebral and renal tissue, respectively [48]. Therefore, our current data confirm the involvement of inflammatory complications in TG/GM-induced renal injury.

Notably, the overall data revealed that combined treatment of GM with TG at a dose rate of 14 mg/kg exerted a more damaging effect than those that received GM with a lower dose of TG (7 mg/kg). These data imply that the occurrence of renal injury in the TG and GM co-treated animals was in a dose-dependent manner. Moreover, according to the presented data in this investigation, oxidative stress is suggested to modulate the damaging effect of the TG and GM combination on the renal tissue, exerting more deleterious effects than their individual treatments. The current findings are in complete agreement with our previous experiments. The tissue injury generated by oxidative stress-inducing agents was promoted when given in combination such as cadmium with piroxicam [41] and cefepime with diclofenac sodium [49].

**Figure 7:**
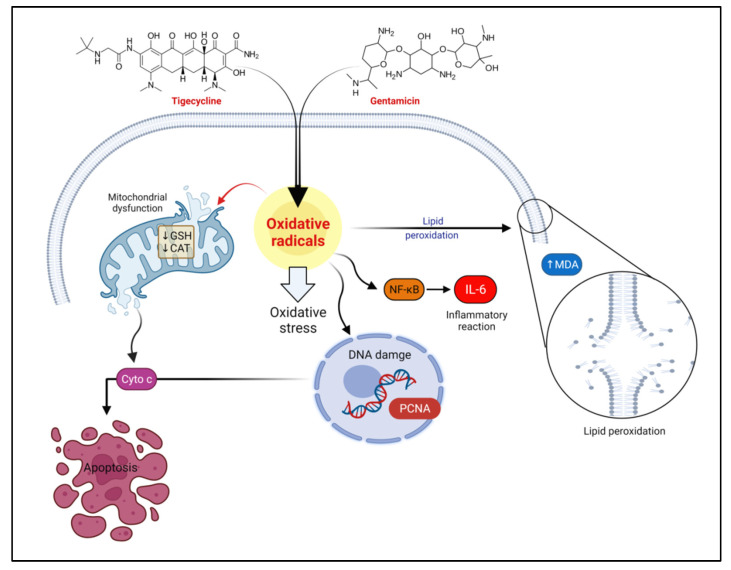
The schematic diagram summarizes the proposed mechanisms behind the tigecycline and gentamicin-potentiated renal injury. CAT, catalase; Cyto c, cytochrome c; GSH, reduced-glutathione; IL-6, interleukin-6; MDA, malondialdehyde; PCNA, proliferating cell nuclear antigen; NF-κB, nuclear factor-kappa B.

Remarkably, when TG and GM were administered in combination (in a dose-dependent rhythm), the employed PCA provided new significant information about their damaging impacts when compared to their individual treatments. The results of the PCA showed that combining TG and GM created a greater divergence in the creatinine, BUN, annexin-V, IL-6, and MDA levels. Thus, the PCA highlights that the TG14+GM group can be distinguished from the TG7+GM group and even from the control, TG7, TG14, and GM groups along the Dim1 with a 71.7% contribution (Figure 6). To our knowledge, this is the first time that the PCA has been used to determine the most important variables behind the pathogenesis of TG and GM-induced kidney injury. However, further studies related to the direct effects of TG and/or GM on mitochondrial function at various regimens are needed to confirm the role of oxidative stress in these pathways. The anticipated mechanisms of TG and GM-potentiated renal injury are depicted in Figure 7.

## 4. Materials and Methods

### 4.1. Drugs

Tigecycline, TG (Tygacil^®^, 50 mg/mL), was obtained from Pfizer Inc., Cairo, Egypt. Gentamicin sulfate, GM (Garamicin^®^, 80 mg/mL) was purchased from Memphis, Cairo, Egypt.

### 4.2. Experimental Animals

The current research was carried out on adult male Wister rats weighing 160–200 g obtained from the Center of Laboratory Animal, Faculty of Veterinary Medicine, Benha University, Egypt. Prior to the experiment, all animals were left for acclimatization for two weeks at a temperature ~25 °C and 12/12 light/dark cycle. Rats received a standard laboratory commercial diet and water ad libitum along the experimental period. Ethical approval from the Ethics Committee of the Faculty of Veterinary Medicine, Benha University for the study protocol and utilization of rats was obtained (approval no. BUFVTM 05-02-21).

### 4.3. Experimental Design

Rats were assigned into six equal groups (five rats each). The first group (control); received saline, i.p., the second (TG7) and third groups (TG14) received TG (7 and 14 mg/kg, i.p., respectively). This regimen was set based on that made by Vergidis et al. [29] with simple modifications. In the fourth group (GM), rats were challenged with GM at a dose rate of 80 mg/kg, i.p. The GM dosage was determined according to Sharma et al. [50] with minor modifications. The fifth group (TG7+GM) of rats were concurrently administrated with GM (80 mg/kg) with TG (7 mg/kg). The sixth group (TG14+GM) of rats in this group were provided with both GM (80 mg/kg) and TG (14 mg/kg). Both drugs were administered for 10 consecutive days as a single dose per day with 2 h intervals.

### 4.4. Sampling

Twenty-four hours after the end of the experiment, rats were euthanized, and blood samples were collected from the retro-orbital plexus. Next, sera were separated at 2000× *g* for 10 min and stored at −20 °C for further biochemical analyses. However, the kidneys were dissected and perfused in ice-cold PBS. The right kidney was preserved in 10% neutral-buffered formalin and assigned for histological examination while a part of the left kidney was stored at −80 °C for oxidative damage determination. Another part was transferred in an isotonic saline for flow cytometry measurements of annexin-V and interleukin-6 (IL-6).

### 4.5. Serum Biochemical Studies

Serum creatinine, blood urea nitrogen (BUN), and glucose were assessed using diagnostic kits purchased from Laboratory Biodiagnostics Co, Cairo, Egypt.

### 4.6. Tissue Oxidative Cascade Evaluation

The kidney tissue was dissected and rinsed by ice-cold PBS (phosphate-buffered saline) consisting of 0.16 mg/mL heparin to separate any RBCs and curd. The tissue was then weighed, and one gram was homogenized by a sonicator homogenizer using a 5 mL buffer solution composed of 50 mM potassium phosphate and 1 mM EDTA (pH 7.5). Afterward, the obtained tissue homogenate was centrifuged in a cooling centrifuge (4000 rpm for 20 min) and then kept at −80 °C. Later, the oxidative status evaluation was conducted by the determination of malondialdehyde (MDA) concentration, catalase (CAT) activity, and reduced-glutathione (GSH) level. All procedures were performed according to the manufacturer’s instructions (Laboratory Biodiagnostics Co.).

### 4.7. Histopathological Examinations

After fixation of the renal tissue in 10% neutral-buffered formalin for 72 h, the tissue was progressively dehydrated in ascending grades of ethyl alcohol and cleared in xylene. Next, all specimens were embedded in Paraplast, cut into 5 μm sections, and then stained with hematoxylin and eosin (H&E) for histological inspection. Furthermore, according to Aydın et al. [51], the alterations of the histopathological parameters of the kidney were graded as follows: no, (−); mild, (+); moderate, (++), and severe (+++) histological changes, respectively. Five random fields at 400× were checked blindly using a light microscope (DM3000, Leica, Germany).

### 4.8. Immunohistochemical Examination

The kidney tissue sections were deparaffinized in xylene and rehydrated using descending grades of alcohol. The antigen retrieval process was performed in 10 mM sodium citrate buffer boiled in a microwave. Then, endogenous peroxidases were blocked using 0.03% hydrogen peroxide for 5 min. Tissue sections were washed gently with PBS and the nonspecific sites were blocked by 5% BSA. Next, the sections were incubated in a humidity chamber with the primary antibody (proliferating cell nuclear antigen; PCNA, Thermo Fisher Scientific, Manor Park, United Kingdom) at a dilution rate of 1:200 for 60 min at 37 °C, followed by washing for 3-times in PBS. After, all slides were incubated for 45 min at 37 °C with the secondary antibody (Envision system labeled polymer reagent; Dako, Kyoto, Japan). The positive brown stained nuclei were visualized by 3,3-diaminobenzidine-substrate chromagen, DAB (Dako, Kyoto, Japan), and counterstained with Mayer’s hematoxylin for 5 s.

### 4.9. Annexin-V-FITC and Interleukin-6 (IL-6) Assay

According to the manufacturer’s procedure, a single-cell suspension from the kidney tissue was used to evaluate the degrees of apoptosis and inflammatory response as previously described by Pathak and Khandelwal [52]. Apoptosis and inflammatory cytokine quantification kits (Biorbyt Ltd., Cambridge, UK) were used. The analysis was performed using a flow cytometer (BD Accuri™ C6, BD Biosciences, San Jose, CA, USA).

### 4.10. Statistical Analysis

Data analyses were performed using one-way analysis of variance (ANOVA) followed by Duncan’s post hoc test using SPSS (Version 21; SPSS Inc., Chicago, IL, USA) to compare the treatment means at a 5% significance level (*p* < 0.05). All values are explicated as the mean and 95% confidence interval. Multivariate principal component analysis (PCA) was performed using ‘Factoextra’, and the ‘FactoMineR’ packages were built in Rstudio under R version 4.0.2.

## 5. Conclusions

Concurrent TG and GM treatment resulted in more profound renal damage (in a dose-dependent modality) than even their individual treatments. The key mechanisms involved in this potentiated toxicity were oxidative stress, inflammatory response, and apoptosis. According to the PCA, creatinine, BUN, annexin-V, IL-6, and MDA all played a role in distinguishing the TG and GM combinations from other groups. Based on this study, prescribing TG with GM should be carried out with caution and prudence to minimize any adverse effects.

## Figures and Tables

**Table 1:** A summary of the renal histopathological changes among the different groups.

	Experimental Groups					
	Control	TG7	TG14	GM	TG7+GM	TG14+GM
Congestion of glomerular tuft	−	−	+	−	−	−
Congestion of intertubular capillaries	−	−	+	−	+	+
Intratubular eosinophilic debris	−	+	+	+	++	+++
Tubular degeneration	−	−	−	+	++	+++

−, no; +, mild; ++, moderate; +++, severe histological changes.

## Data Availability

Upon request, the data available within article and Supplementary Materials are obtainable from the corresponding authors.

## Supplementary Materials

The following supporting information can be downloaded at: https://www.mdpi.com/article/10.3390/ph15060736/s1, Figure S1: Alternative images for renal histopathology after TG and/or GM treatment.

## Abbreviations

BUN: blood urea nitrogen; CAT, catalase; FDA, Food and Drug Administration; GM, gentamicin; GSH, reduced-glutathione; H_2_O_2_, hydrogen peroxide; IL-6, interleukin-6; MDA, malondialdehyde; NO, nitric oxide; O_2_^•−^, superoxide anion; OH^•^, hydroxyl radical; PCA, principal component analysis; PCNA, Proliferating cell nuclear antigen; TG, tigecycline; NF-κB, Nuclear factor-kappa B.
