# Supplementary material for: Tigecycline and Gentamicin-Combined Treatment Enhances Renal Damage: Oxidative Stress, Inflammatory Reaction, and Apoptosis Interplay
Source: Pharmaceuticals (Basel). 2022 Jun 10;15(6):736. doi: 10.3390/ph15060736 (PMC9228782; doi:10.3390/ph15060736)
Supplement: Supplementary file 1 [file pharmaceuticals-15-00736-s001.zip › pharmaceuticals-1699094-supplementary final 2.pdf]

pharmaceuticals

MDPI

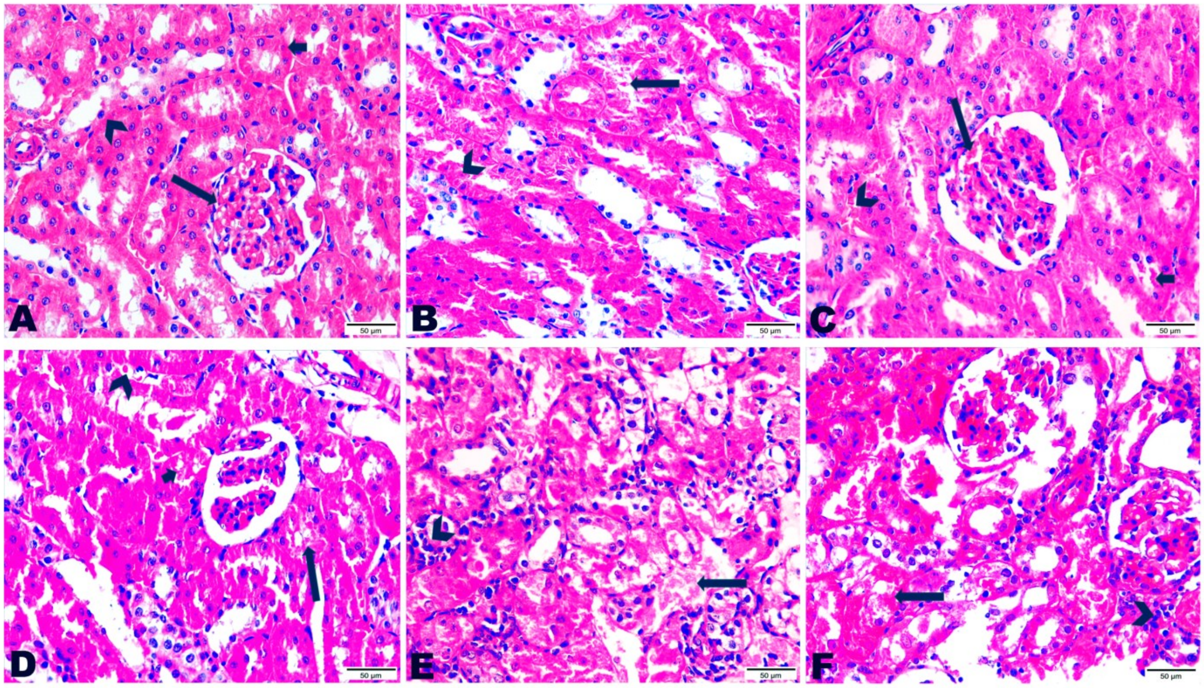

Figure S1. Photomicrographs presented histopathological changes of kidney sections between examined groups. (**A**)**:** Control group. (**B**)**:** TG7-treated group. (**C**)**:** TG14-treated group. (**D**)**:** GM-treated group. (**E and F**)**:** TG7 + GM and TG14 + GM-treated groups, respectively. (H&E stain, x400 magnification, scale bar = 50μm).
